# High-Intensity Interval Training Improves Inhibitory Control and Working Memory in Healthy Young Adults

**DOI:** 10.5114/jhk/194498

**Published:** 2025-05-29

**Authors:** Tian Yue, Hanghang Su, Ming-Yang Cheng, Yan Wang, Ke Bao, Fengxue Qi

**Affiliations:** 1 Key Laboratory of Sport Training of General Administration of Sport of China, Beijing Sport University, Beijing, China.; 2 Sports, Exercise and Brain Sciences Laboratory, Sports Coaching College, Beijing Sport University, Beijing, China.; 3 School of Psychology, Beijing Sport University, Beijing, China.; 4 School of Sports Medicine and Rehabilitation, Beijing Sport University, Beijing, China.

**Keywords:** endurance training, moderate exercise, continuous exercise

## Abstract

High-intensity interval training (HIIT) has emerged as a time-efficient mode of exercise. However, there is limited evidence that HIIT improves inhibitory control and working memory (WM) more than moderate-intensity continuous exercise (MICT). This study aimed to explore the effects of HIIT, moderate-intensity interval exercise (MIIT), and MICT on inhibitory control and WM in healthy adults. Twenty-five healthy college students (21.04 ± 2.44 years; 14 females) were recruited to complete HIIT, MIIT, MICT, and a resting session (CON) for 15 min in a randomized crossover design. The HIIT protocol comprised three 3-min bouts at 90% of the maximum heart rate (HR_max_) with 2 min of active recovery at 70% HR_max_. The MIIT protocol comprised three 3-min bouts at 70% HR_max_ with 2 min of active recovery at 50% HR_max_. A volume-matched MICT protocol was applied at 70% HR_max_. The Stroop and 2-back tasks were used to evaluate inhibitory control and WM in post-trials, respectively. Response times (RTs) of the Stroop task significantly improved on the congruent condition in the HIIT session compared to the CON session (p = 0.004, Cohen’s d = 0.64), on the incongruent condition in the HIIT session compared to MICT (p = 0.049, Cohen’s d = 0.42) and CON (p = 0.023, Cohen’s d = 0.49) sessions, and on the neutral condition in the HIIT session compared to MIIT (p = 0.029, Cohen’s d = 0.47) and CON (p = 0.012, Cohen’s d = 0.55) sessions. Hits in the 2-back task increased significantly following HIIT compared to MIIT (p = 0.041, Cohen’s d = 0.43), MICT (p = 0.02, Cohen’s d = 0.5), and CON (p = 0.006, Cohen’s d = 0.6). We concluded that a single bout of HIIT effectively improved inhibitory control and WM in healthy adults. These findings support the practical implication of HIIT being beneficial within a short time for enhancing inhibitory control and WM in clinical populations.

## Introduction

Inhibitory control refers to the capacity to regulate attention, manage emotions, alter automatic behaviors, and direct thoughts towards stimuli that are relevant to a given task, while overcoming internal impulses and external attraction (Best, 2010). Human behavior can be influenced by environmental stimuli, but improving inhibitory control can make it easier to regulate behavior and, thus, avoid making poor decisions. Working memory (WM) is characterized by the capacity to transiently store and manipulate information required for complex tasks (reasoning, comprehension, and learning) (Baddeley, 2010). Updating is defined as the process of reorganizing information in WM storage (Morris and Jones, 1990). This process is intricately connected to advanced cognitive capabilities, including fluid and crystallized intelligence (Friedman et al., 2006), reading comprehension (Carretti et al., 2005), and mathematical calculations (Deschuyteneer et al., 2006; Gathercole et al., 2004). The frontal, parietal, and temporal lobes are closely associated with inhibitory control and WM, and these brain regions represent the functional division of work and collaboration (Baddeley, 2003; Hampson et al., 2006; Munakata et al., 2011). In particular, the prefrontal cortex (PFC) is the most important brain region for inhibitory control and WM (Alvarez and Emory, 2006; Munakata et al., 2011). The PFC constitutes a complex network of neocortical regions engaged in the reception and integration of signals from virtually all cortical sensory and motor systems, as well as numerous subcortical entities. It plays a pivotal role in the mediation of inhibitory control and WM functions (Miller and Cohen, 2001).

Exercise can modulate brain plasticity and improve cognitive function (Chen et al., 2011). Moderate-intensity continuous training (MICT) and high-intensity interval training (HIIT) are widely used because they are readily adapted to and have few adverse effects. MICT is exercise characterized by uninterrupted and moderate-intensity activity (e.g., 64%–76% of the maximum heart rate) (Garber et al., 2011). HIIT is interval exercise characterized by alternating sequences of high-intensity physical exertion and periods of rest or moderate activity (Dolan et al., 2024; Wisløff et al., 2007). Moderate-intensity interval training (MIIT) is a subcategory of interval exercise involving moderate intensity. Some studies have found that just one session of HIIT can enhance inhibitory control and WM. HIIT stands out as a highly efficient exercise regimen, particularly beneficial for enhancing the brain-derived neurotrophic factor (BDNF) (Saucedo Marquez et al., 2015), a biomarker associated with inhibitory control and WM (Slusher et al., 2018). Previous studies showed that acute HIIT promoted PFC activation and oxygenation (Kao et al., 2017; Lambrick et al., 2016), which attenuated inhibitory control and WM decline (Ai et al., 2021). Synaptic plasticity, delineated as the ability of the nervous system to adjust interneuronal communication intensity (Citri and Malenka, 2008), emerges as a pivotal mechanism within this framework. Notably, a session of HIIT lasting 20 min has proven to be more effective than moderate-intensity continuous exercise (MICT) in improving motor cortex neuroplasticity, highlighting a significant potential for HIIT in cognitive enhancement strategies (Andrews et al., 2020). Acute HIIT may enhance synaptic plasticity, thus benefiting inhibitory control and WM (Chang et al., 2012). However, some studies do not support the notion that HIIT can improve inhibitory control and WM. A meta-analysis revealed more pronounced enhancements in cognitive function occurring within 11 to 20 min subsequent to acute exercise sessions (Chang et al., 2012). The null effect of HIIT in some studies might be attributed to the point at which cognitive assessment took place or the long duration of HIIT (Schwarck et al., 2019). Greater neuromuscular fatigue and lower parasympathetic tone during HIIT might attenuate the magnitude of improvement on reaction time (Dupuy et al., 2018).

Studies have shown that MICT improves inhibitory control and WM in healthy adults (Chang et al., 2017; Kao et al., 2020; Ligeza et al., 2018). An event-related potential study found that MICT improved inhibitory control and increased the N2 component, a neural marker of inhibitory control, compared with the resting condition (Ligeza et al., 2018). Compared with severe-intensity HIIT of long duration, MICT induced an increase in cortical oxygenation and stable levels of blood lactate that could benefit inhibitory control and WM (Ligeza et al., 2018). Besides, Ludyga et al. (2019) demonstrated that MIIT improved inhibitory control up to 60 min after MIIT cessation in male adolescents, but HIIT failed to promote inhibitory control. A decreased global cerebral blood flow was observed during high-intensity exercise (Smith and Ainslie, 2017), which might be a possible reason why HIIT elicited no changes in inhibitory control in adolescents. However, some studies also found that acute MICT did not improve inhibitory control and WM (Ballester-Ferrer et al., 2022; Li et al., 2014; Yamazaki et al., 2018). Another study revealed that improvements in WM function after acute MICT depended on baseline WM (Yamazaki et al., 2018). In addition, a scarcity of studies have explored the effects of HIIT versus MIIT on inhibitory control and WM in healthy adults. A study by Loprinzi et al. (2019) showed that, relative to low- and moderate-intensity exercise, high-intensity exercise would impair WM. Although HIIT is a more time-efficient mode of exercise, there is currently insufficient evidence that it improves inhibitory control and WM more than MICT. Therefore, HIIT should not be regarded as a replacement for MICT. Different exercise types, exercise intensity and populations in previous studies have led to mixed results and were inconclusive regarding the effect of HIIT on inhibitory control and WM (Pontifex et al., 2019). Despite the significant focus on exercise intensity, the specific effect of the type of exercise (e.g., continuous vs. intermittent) on inhibitory control and WM in healthy adults remains unexplored. Furthermore, many studies involved cross-sectional comparisons between HIIT and other exercises, and only few longitudinal studies have been undertaken.

Therefore, we conducted a randomized crossover trial to evaluate the impact of HIIT on inhibitory control and WM. Specifically, in this study, we contrasted the effect of a single session of HIIT, MIIT, and MICT on inhibitory control and WM using the Stroop task and the 2-back task with healthy young adults. Our hypothesis posited that HIIT would improve inhibitory control and WM to a greater extent than MIIT and MICT in healthy young adults.

## Methods

### Participants

Twenty-seven healthy college students (13 males, 14 females) with exercise experience were recruited. Two male participants dropped out during the experiment because of previous injuries requiring surgery. Twenty-five participants (11 males, 14 females) completed the experiment and their demographic characteristics are shown in Table 1. Basic health status and health-screening questionnaires confirmed that participants were free of respiratory and cardiovascular diseases. Each participant exhibited either normal or corrected-to-normal vision and was identified as right-handed based on the Edinburgh Handedness Inventory. None of the participants had color-blindness/weakness, a sports injury in the past 6 months, and a personal or family history of mental health difficulties. All participants provided their written informed consent before taking part in the study. This study was approved by the Ethics Committee for Human Experiments of the Beijing Sport University (approval code: 2021066H; approval date: 28 April 2021) and conformed to the Declaration of Helsinki.

**Table 1:** Demographic information for all participants, n = 25 (14 females); data presented as mean (SD).

Age (years)	21.04 (2.44)
Body height (m)	1.73 (0.10)
Body mass (kg)	65.78 (13.91)
BMI (kg/m^2^)	21.83 (3.10)
HR_max_ (bpm)	182.32 (8.00)
MPO (W)	204.36 (2.20)

Note: BMI, body mass index; HR_max_, maximum heart rate; MPO, maximum power output

### Procedures

**Figure 1:**
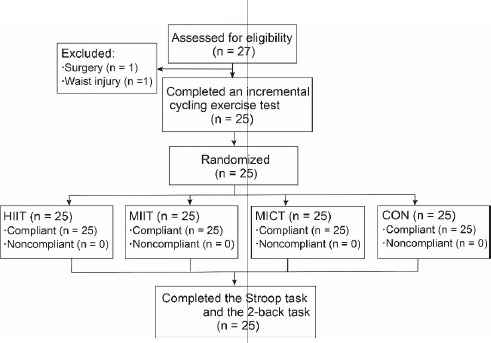
The experimental procedures. Note: HIIT, high-intensity interval training; MIIT, moderate-intensity interval training; MICT, moderate-intensity continuous training; CON, resting control; HR, heart rate

The randomized crossover design was adopted in this study. Twenty-five participants randomly received HIIT, MIIT, MICT and resting control intervention (CON) with an interval of at least one week in between. When participants came to the laboratory for the first time, their informed consent was received, and then health-screening questionnaires and the Edinburgh Handedness Inventory were completed prior to testing. Participants' body height and mass were recorded to determine their body mass index (BMI). The incremental cycling exercise test to exhaustion on a cycle ergometer was used to determine the participants’ maximum heart rate (HR_max_) and maximum power output (MPO). At least 72 h after the incremental cycling exercise test, the exercise protocols of HIIT, MIIT, MICT, and CON were performed randomly at the same time on four different days (Andrews et al., 2020; Kao et al., 2020). Each session of exercise included a warm-up lasting 2 min, followed by 15 min dedicated to cycling, concluding with an 8-min rest interval, all conducted under identical laboratory conditions. An 8-min rest interval was sufficient for participants to recover their baseline HR, avoiding the interference of the HR on executive function (EF) assessment. Then, the Stroop task and the 2-back task were immediately undertaken (Figure 1).

### Incremental Cycling Exercise Test

Participants were asked to ensure they had sufficient sleep and to eat normally. They were also required to avoid vigorous physical activity and consumption of alcohol for at least 24 h before the test. Caffeine was prohibited for at least 12 h before the test and each intervention day.

All participants completed the incremental cycling exercise test to determine their maximum HR and MPO (W) to enable the research team to adjust the workload for HIIT, MIIT, and MICT. The warm-up workload and increment workload per minute were determined by pre-experiments. Participants engaged in a 3-min warm-up on a cycle ergometer (Ergoselect 100k, Ergoline, Germany), with resistance set to 50 W for females and 75 W for males, at a cadence of 60 revolutions per minute (RPM). The load was then automatically increased by 25 W per minute until volitional exhaustion of participants. The HR, MPO and rating of perceived exertion (RPE) data of participants were collected during the test. The criteria for determining the volitional exhaustion of participants were as follows: (1) pedaling cadence fell below 60 RPM for more than 5 s; (2) RPE ≥ 17; and (3) HR reached 206.9 − 0.67 × age ± 10 (Gellish et al., 2007; Strasser et al., 2016; Suruagy et al., 2017).

### Exercise Interventions

In each session, the exercise workload was adjusted to ensure the target HR was reached according to the corresponding relationship between the HR and MPO obtained in the incremental cycling exercise test. We determined the optimal HIIT and MIIT protocols through multiple pre-experiments that would ensure appropriate HRs based on the required workload in work and recovery sessions. The HIIT protocol involved cycles of 3 min at 90% HR_max_ interspersed with 2 min at 70% HR_max_, spanning a total duration of 15 min (Andrews et al., 2020; Follador et al., 2018; Garber et al., 2011). The MIIT protocol involved alternating between 3 min of cycling at 70% HR_max_ and 2 min of cycling at 50% HR_max_, for a total duration of 15 min (Garber et al., 2011; Jiménez-García et al., 2021). The MICT protocol consisted of 15 min of cycling at 70% HR_max_ (Garber et al., 2011; Tian et al., 2021). For the rest (CON) condition, participants were instructed to sit on the cycle ergometer for 15 min, but were instructed not to pedal. Each exercise session was initiated with a warm-up lasting 2 min at 50 W, and concluded with a rest interval of 8 min.

### Stroop Task

The Stroop task was used to assess inhibitory control. Stimulus words consisted of four color names (red, green, blue, yellow) rendered in Chinese characters and exhibited on a 17-inch computer screen with a black background. At a comfortable reading distance, the size of the words was 2.5 cm. The task comprised three conditions. In the congruent condition, four distinct words were displayed to participants, each written in a color that matched the word's semantic meaning (e.g., the word “blue” was written in blue). Four buttons on a keyboard represented the four colors (red-D, green-F, blue-J, yellow-K). Participants were directed to rapidly and accurately determine the color of each word, and to indicate their response by pressing the corresponding button for the answer. In the neutral condition, four “X” shapes were presented in red, green, blue, and yellow, and participants were asked to respond by pressing the respective answer button to indicate the color as quickly and accurately as possible. In the incongruent condition, the colors of the stimulus words were different from the meaning of the words (e.g., the word “blue” was written in green). Participants were asked to press the corresponding button of the color in which the word was written as quickly and accurately as possible. Each word stimulus was displayed on the screen for 500 ms and the inter-stimulus interval was 2 s. For each condition, stimulus words were displayed in a random sequence. Each condition included 48 stimulus words and this was conducted over three separate trials. The total experiment duration was 4 min. The accuracy (i.e., the correct number of tasks) and response times (RTs) were identified as behavioral measures for this task.

### 2-Back Task

A 2-back task was programmed to assess WM. The target letters A–J were used as stimuli presented randomly on a 17-inch computer screen, appearing in black with a white background. The size of the stimuli was 1 cm at a comfortable reading distance. Participants were required to determine if the present stimulus was identical to the one shown two steps prior in the sequence. The “V” button and “N” button represented congruent (matching) and incongruent (not matching), respectively. Participants were guided to answer both rapidly and precisely. Stimuli in the task were displayed for 500 ms each, with a consistent inter-stimulus gap of 1500 ms. The button was pressed 140 times in total, and the ratio of match to mismatch was 1:2. The total experiment duration was 7 min. Hits (correct responses), misses (incorrect responses), false alarms, correction rejections, and RTs were identified as behavioral measures for this task.

### Statistical Analysis

RTs shorter than 200 ms or longer than 1500 ms were excluded from the analysis of RTs. In addition, RTs that were greater than 3 standard deviations of the individual mean value were excluded. The Shapiro-Wilk test was used to examine the normality of the dataset. Data that were not normally distributed were transformed into natural logarithms (ln) prior to the statistical analysis.

For the Stroop task, two-way repeated measures analyses of variance (ANOVAs) were conducted with session (HIIT, MIIT, MICT, and CON) and condition (congruent, incongruent, and neutral) serving as within-subject factors for the dependent variables (RTs and accuracy). For the 2-back task, one-way repeated measures ANOVAs with the within-subject factors (session: HIIT, MIIT, MICT, and CON) were conducted for the dependent variables (hits, misses, false alarms, correction rejections, and RTs). In instances where the Mauchly’s test revealed sphericity violations, the Greenhouse-Geisser correction was employed to adjust the degrees of freedom. The Fisher’s least significant difference post hoc test was used to identify where differences occurred.

Partial Eta Squared (ηp2) was computed for ANOVA comparisons, and effect sizes were evaluated as small (≥ 0.01), moderate (≥ 0.059), or large (≥ 0.138) (Bakeman, 2005). Cohen’s *d* values were determined for pairwise comparisons, categorizing the magnitude of effect sizes as small (≥ 0.2), medium (≥ 0.5) or large (≥ 0.8). Data were presented as the mean ± standard deviation, with statistical significance set at *p* < 0.05. All statistical analyses were performed using SPSS version 26 (IBM, Armonk, NY, USA). Graphs were created using GraphPad Prism (GraphPad Software 8.0.2).

## Results

### Psycho-Physiological Measures

**Figure 2:**
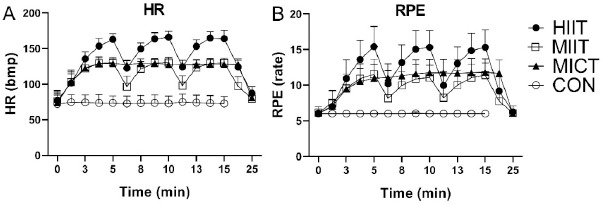
A. Mean heart rate (beats per minute) during exercise and recovery. B. Mean RPE during exercise and recovery. Mean heart rate and RPE in HIIT were significantly higher than in the other conditions. Mean heart rate and RPE in MIIT and MICT were higher than in CON. Note: HIIT, high-intensity interval training; MIIT, moderate-intensity interval training; MICT, moderate-intensity continuous training; CON, resting control; HR, heart rate; RPE, rating of perceived exertion

HR and RPE values during exercise and immediately following exercise cessation are shown in Figure 2. HR and RPE values in HIIT were significantly higher than in the other conditions. HRs in MIIT and MICT were similar, but significantly higher than in CON. In MICT, the RPE was slightly higher than in MIIT. The HR and RPE results showed that the target HR was achieved in the different exercises.

### Stroop Task

**Figure 3:**
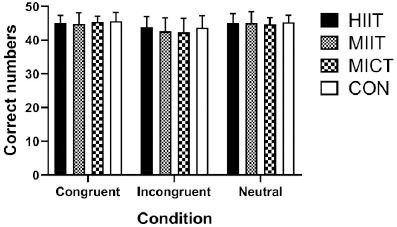
Results of different exercise and resting trials on Stroop task accuracy. Note: HIIT, high-intensity interval training; MIIT, moderate-intensity interval training; MICT, moderate-intensity continuous training; CON, resting control

For accuracy, the repeated measures ANOVA showed a significant main effect of condition (*F*1.481, 35.535 = 27.154, *p* < 0.0001, ηp2 = 0.531), but no significant interaction between the session and condition (*F*3.887, 93.290 =7.455, *p* = 0.321, ηp2 = 0.047), and no significant main effect of the session (*F*2.202, 52.858 = 0.846, *p* = 0.445, ηp2 = 0.034) (Figure 3).

**Figure 4:**
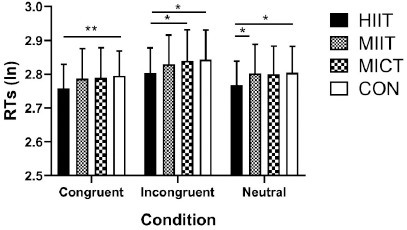
Results of different exercise and resting trials on RTs in the Stroop task. Note: HIIT, high-intensity interval training; MIIT, moderate-intensity interval training; MICT, moderate-intensity continuous training; CON, resting control; RTs, response times. * statistically significant at p < 0.05,** statistically significant at p < 0.01

For RTs, the repeated measures ANOVA exhibited a significant main effect of the session (*F*2.093, 50.229 = 3.577, *p* = 0.033, ηp2 = 0.13) and extremely significant effect of the condition (*F*1.284, 30.822 = 59.591, *p* < 0.0001, ηp2 = 0.713). There was no significant interaction between the session and condition interaction (*F*6, 144 = 0.805, *p* = 0.568, ηp2 = 0.032). In the congruent condition, post hoc analysis revealed that participants were significantly faster in the HIIT session compared with the CON session (*p* = 0.004, Cohen’s *d* = 0.64). In the incongruent condition, participants were significantly faster in the HIIT session compared with the MICT (*p* = 0.049, Cohen’s *d* = 0.42) and CON (*p* = 0.023, Cohen’s *d* = 0.49) sessions. In the neutral condition, after the HIIT session, participants were significantly faster compared with the MIIT (*p* = 0.029, Cohen’s *d* = 0.47) and CON (*p* = 0.012, Cohen’s *d* = 0.55) sessions (Figure 4).

### 2-Back Task

**Figure 5:**
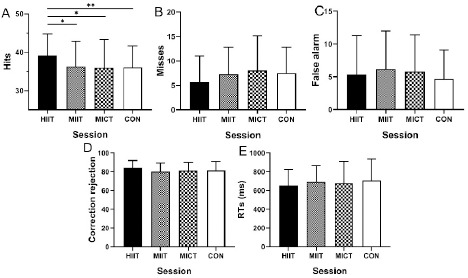
Results of different exercise and resting trials on A. Correct responses (‘hits’) in the 2-back task. B. Incorrect responses (‘misses’) in the 2-back task. C. False alarms in the 2-back task. D. Correction rejections in the 2-back task. E. RTs of accuracy (i.e., hits) in the 2-back task. Note: HIIT, high-intensity interval training; MIIT, moderate-intensity interval training; MICT, moderate-intensity continuous training; CON, resting control; RTs, response times. * statistically significant at p < 0.05,** statistically significant at p < 0.01

The repeated measures ANOVA revealed a significant effect of the session for accurate responses (‘hits’) (*F*3, 72 = 2.836, *p* = 0.044, ηp2 = 0.106). Multiple comparisons showed a significant difference for the HIIT session in comparison with the MIIT (*p* = 0.041, Cohen’s *d* = 0.43), MICT (*p* = 0.02, Cohen’s *d* = 0.5), and CON (*p* = 0.006, Cohen’s *d* = 0.6) sessions. Misses, false alarms, correction rejections, and RTs did not differ between intervention sessions (all values of *p* > 0.05) (Figure 5).

## Discussion

The current study aimed to investigate the effect of HIIT on inhibitory control and WM compared with MIIT and MICT in healthy college students. Considering our hypothesis, a single bout of HIIT improved inhibitory control as shown in the Stroop task. It furthermore increased the number of accurate responses (‘hits’) in the WM task. This discovery highlights the crucial role of exercise intensity in improving inhibitory control and WM in college students.

The present findings align with previous results which showed that acute HIIT improved inhibitory control and WM (Alves et al., 2014; Cooper et al., 2016; Tsukamoto et al., 2016; Wilke, 2020). Alves et al. (2014) reported that a single bout of HIIT shortened RTs in the Stroop task in healthy middle-aged individuals. A randomized crossover study by Cooper et al. (2016) showed that healthy adolescents’ RTs for the Stroop task were more rapid immediately following HIIT compared with other exercise regimes, although their task accuracy was not affected. Bahdur et al. (2019) observed notable enhancements in RTs and accuracy in the Stroop task immediately after an acute HIIT bout in recreationally active college students. Acute HIIT has the possibility of improving inhibitory control in young and middle-aged individuals. Moreover, Wilke (2020) found that acute HIIT improved WM in college students. Probable mechanisms of action include the increase in the BDNF brought about by HIIT. The BDNF is a biomarker associated with inhibitory control and WM, and high-intensity exercise results in larger increases in the BDNF compared with low-intensity exercise (Knaepen et al., 2010; Slusher et al., 2018). Winter et al. (2007) found that HIIT improved learning performance and elicited greater BDNF and catecholamine levels compared with MICT in healthy adults, with the BDNF and catecholamine acting as mediators between exercise intensity and EF (Winter et al., 2007). HIIT may elicit beneficial effects on RTs in the Stroop task accompanied by an increased BDNF, insulin-like growth factor-1 (IGF-1), vascular endothelial growth factor, and blood lactate concentration in healthy young males (Kujach et al., 2020). Martínez-Díaz et al. (2020) observed that a single bout of HIIT improved WM performance with a significant response on the BDNF and cortisol levels, and the improvement could be sustained through 30 min of recovery after the exercise. While HIIT may lead to progressive fatigue and heightened central nervous system activation, it also stimulates the production of the BDNF and IGF-1. These biomolecules are beneficial for neurogenesis and synaptic plasticity in the brain, consequently enhancing inhibitory control (Kujach et al., 2020).

Improvement of inhibitory control and WM induced by HIIT may be attributed to the PFC activation. The PFC participates in the process of judgment, planning, and decision-making, all of which play a significant role in inhibitory control and WM (Funahashi, 2001; Li et al., 2014). Previous studies also proved that acute HIIT affected the PFC by increasing PFC activation and oxygenation (Kao et al., 2018; Lambrick et al., 2016). Burin et al. (2020) demonstrated that after a virtual-based HIIT intervention, healthy young adults executed the Stroop task rapidly and the left dorsolateral PFC activity, tested by functional near-infrared spectroscopy, also coherently increased. Kujach et al. (2018) found that acute HIIT enhanced inhibitory control in young adults, probably mediated by increased activation of the PFC. Overall, the preferential enhancement of PFC-dependent EF (e.g., inhibitory control and WM) by HIIT may be the mechanism for the improvement of inhibitory control and WM. Additionally, it was noted that psychological variables, particularly arousal levels, were elevated following HIIT as opposed to MICT. This increase in arousal might be crucial in maintaining the exercise-induced enhancement of inhibitory control (Tsukamoto et al., 2016). Cooper (1973) posited that acute exercise activated the hypothalamus and the brain stem, stimulating the sympathoadrenal system and leading to elevated levels of brain neurotransmitters, including dopamine and norepinephrine (Cooper, 1973). Supporting this view, McMorris et al. (2011) proposed that heightened neurotransmitter levels could enhance cognitive function. Theoretically, the increase in catecholamine concentrations in the brain after engaging in HIIT is expected to improve inhibitory control and WM.

From the perspective of energy metabolism, lactate could serve as a mediator for the positive impact of acute exercise on inhibitory control and WM, functioning both as a source of energy and a signaling molecule (Xue et al., 2022). Tsukamoto et al. (2016) found that, in comparison to the MICT group, the HIIT group showed significant improvements in the RTs of the Stroop task and exhibited notably higher blood lactate levels during the 30-min recovery period post-exercise. Kujach et al. (2020) suggested that HIIT-induced blood lactate could be a contributing factor to enhanced inhibitory control. In contrast, a recent investigation revealed that despite significant differences in blood lactate concentrations between HIIT and MICT groups, these concentrations did not correlate with measures of inhibitory control, including accuracy and RTs (Zhu et al., 2021). Consequently, the link between lactate levels and the functions of inhibitory control and WM warrants further exploration.

Evidence from various studies indicates that exceeding a certain intensity threshold in high-intensity exercise cannot improve inhibitory control and WM performance (Cooper et al., 2016; Kamijo et al., 2007; Ligeza et al., 2018; McMorris and Hale, 2012). For example, diverging from the present study, Ligeza et al. (2018) managed metabolic variations associated with exercise intensity by employing Ventilatory Threshold Determination, and conducted HIIT of longer duration (24 min vs. 15 min). Discrepancies between the current study and prior research could be attributed to the specific exercise protocols and cognitive tasks employed. The impact of exercise on WM may be contingent upon the characteristics of the exercise regimen (i.e., intervention type, intensity, time, and recovery) and sample characteristics (i.e., age, gender, and fitness level) (Ai et al., 2021). Cooper et al. (2016) reported no effect of short-time sprint-based HIIT on WM in adolescents, while a recent study (Tottori et al., 2019) suggested that four weeks of HIIT had positive effects on WM in children aged 8–12 years. It seems that a long-term intervention may have more benefits on WM, taking into account that a single bout of HIIT could not improve WM in healthy middle-aged adults (Alves et al., 2014). The null effect of the study by Alves et al. (2014) might be ascribed to fatigue, both peripheral and central, induced by HIIT in participants of late middle age and older (Alves et al., 2014; Tsai et al., 2021). Together, these studies suggest that individual differences should be considered when exploring the effects of HIIT mode on WM.

The current study also differed from previous work in that we found no improvements in inhibitory control and WM induced by acute MICT and MIIT. A study conducted by Kao et al. (2018) revealed that both HIIT and MICT interventions yielded comparable beneficial outcomes on the RTs for the Flanker task. Those authors concluded that HIIT and MICT could improve inhibitory control compared with rest conditions in healthy adults (Kao et al., 2018). In contrast, findings from our study demonstrated that acute MICT did not improve inhibitory control in healthy college students. The difference between the current study and that of Kao et al. (2018) might be because of the assessment of inhibitory control (Stroop task vs. Flanker task), modality of exercise (cycle ergometer vs. treadmill) and intervention duration (15 min vs. 20 min). Tsukamoto et al. (2016) reported the same improvement in inhibitory control immediately after HIIT and MICT in healthy adults. However, they pointed out a decline in the MICT-induced enhancement of inhibitory control back to baseline levels within 30 min following exercise, while the gains in inhibitory control from HIIT were maintained throughout the recovery period (Tsukamoto et al., 2016). The inhibitory control assessment method (Stroop task) and exercise modality (cycle ergometer) in the Tsukamoto et al.’s (2016) study were the same as in the present research, but the MICT duration in their study was 40 min. There might be a time effect on the inhibitory control performance induced by MICT, which created a disadvantage for MICT (time-consuming) compared with HIIT. Although the study that found improvements in inhibitory control induced by MIIT was undertaken with adolescents, possibly leading to a mismatch between the findings of the current study and previous work (Ludyga et al., 2019).

However, the lack of impact from acute MICT on inhibitory control and WM aligns with previous research findings. In research involving a similar population (college students) and a cognitive task (Stroop task), only acute HIIT positively impacted inhibitory control, whereas there was no improvement in inhibitory control after acute MICT (Ballester-Ferrer et al., 2022). Some studies using a similar cognitive task (2-back task) also found no enhancements in WM following acute MICT sessions (Li et al., 2014; Yamazaki et al., 2018). Interestingly, a single bout of yoga, as opposed to cycling, was shown to improve WM, indicating the influence of the exercise type on the acute effects on WM (Gothe et al., 2013). In addition, prior investigations that observed WM improvements subsequent to a single MICT session typically employed more complex WM tasks, such as the modified Sternberg test and the facial N-back task (Pontifex et al., 2009; Weng et al., 2015). In contrast, the current study and previous studies that used a simple n-back task found no improvement in WM after acute MICT (Li et al., 2014; Yamazaki et al., 2018). Furthermore, the influence of a single session of MICT on behavioral outcomes and brain activation may not be synchronized. Li et al. (2014) found that although acute MICT did not improve behavioral performance in the 2-back task, it did prompt increased activation in the right middle prefrontal gyrus, the right lingual gyrus, and the left fusiform gyrus, alongside decreased activation in the anterior cingulate cortexes, the left inferior frontal gyrus, and the right paracentral lobule. That study indicated that a single bout of MICT could benefit WM at a macro-neural level (Li et al., 2014).

Several limitations in the present study should be mentioned. First, whether the results are applicable to other populations needs to be further studied because our research focused only on healthy adults. Second, only the RTs in the Stroop task exhibited a significant main effect of sessions (HIIT, MIIT, MICT, and CON). Although previous studies have shown accuracy is not a sensitive measure of cognitive function related to physical exercise (Ellemberg and St-Louis-Deschênes, 2010; Liu et al., 2020), future research could use complex tests to explore changes in accuracy induced by exercise. Third, intervention duration could potentially affect the benefits of exercise on inhibitory control and WM. Consequently, further studies are needed to investigate the influence of long-term HIIT on inhibitory control and WM, given the single bout of HIIT conducted in this study. Finally, biochemical variables should be collected in future studies to further investigate the mechanisms that may explain the association between HIIT and enhancements in inhibitory control and WM.

## Conclusions

HIIT is well tolerated, and its single bout can improve inhibitory control and WM in healthy adults. The present findings are significant for formulating exercise interventions for young adults to improve their inhibitory control and WM in daily study and work. Further research is warranted to elucidate the long-term HIIT effects on inhibitory control and WM in middle-aged and older adults in the context of age-related cognitive disorders.
